# Chronic kidney disease is a main confounding factor for 25-vitamin D measurement

**DOI:** 10.1590/2175-8239-JBN-2019-0053

**Published:** 2019-09-26

**Authors:** Hanna Karla Andrade Guapyassú Machado, Carolina Steller Wagner Martins, Vanda Jorgetti, Rosilene Motta Elias, Rosa Maria Affonso Moysés

**Affiliations:** 1 Universidade de São Paulo, Divisão de Nefrologia, São Paulo, SP, Brasil.; 2 Hospital Samaritano, São Paulo, SP, Brasil.; 3 Universidade Nove de Julho, São Paulo, SP, Brasil.

**Keywords:** Vitamin D, Immunoassay, Chronic Kidney Disease-Mineral and Bone Disorder, Renal Insufficiency, Chronic, Vitamina D, Imunoensaio, Distúrbio Mineral e Ósseo na Doença Renal Crônica, Insuficiência Renal Crônica

## Abstract

**Background:**

Current guidelines recommend assessment of 25-vitamin D status in patients with chronic kidney disease (CKD). Although significant differences among assays have been described, the impact of CKD on this variability has never been tested.

**Methods:**

We tested the variability between two 25-vitamin D assays in patients with CKD (eGFR < 60 mL/min/1.73m^2^) who had consecutive 25-vitamin D measurements in 2015 (Assay 1 - Diasorin LIASON 25 TOTAL - D assay^®^) and 2016 (Assay 2 - Beckman Coulter Unicel Xl 800^®^). The cohort consisted of 791 adult patients (122 with normal renal function and 669 with CKD - 33, 30, and 37% in stages 3, 4, and 5 on dialysis, respectively).

**Results:**

Levels of 25-vitamin D were lower and the prevalence of hypovitaminosis D using assay 1 was higher than using assay 2 in patients with CKD, regardless of similar levels of calcium, phosphate, and parathyroid hormone. As kidney function decreased, the percentage of disagreement between the assays increased.

**Conclusion:**

There is a noteworthy variability between assays in patients with CKD such that the diagnosis of hypovitaminosis D is modified. The mechanism behind this result is still unclear and might be due to a possible interference in the analytical process. However, the clinical significance is unquestionable, as the supplementation of vitamin D can be erroneously prescribed to these patients.

## Resumo

**Antecedentes:**

As diretrizes atuais recomendam a avaliação do estado da 25-hidroxivitamina D em pacientes com doença renal crônica (DRC). Embora significativas diferenças entre os ensaios tenham sido descritas, o impacto da nesta variabilidade DRC nunca foi testado.

**Métodos:**

Testamos a variabilidade entre dois ensaios de 25-hidroxivitamina D em pacientes com DRC (TFGe < 60 mL/min/1,73 m^2^) que realizaram medidas consecutivas de 25-hidroxivitamina D em 2015 (Ensaio 1 - Diasorin LIASON 25 TOTAL - D assay^®^ ) e 2016 (Ensaio 2 - Beckman Coulter Unicel Xl 800^®^). A coorte consistiu de 791 pacientes adultos (122 com função renal normal e 669 com DRC - 33, 30 e 37% nos estágios 3, 4 e 5 em diálise, respectivamente).

**Resultados:**

Os níveis de 25-hidroxivitamina D foram menores e a prevalência de hipovitaminose D foi maior utilizando o ensaio 1 do que com o ensaio 2 em pacientes com DRC, independentemente dos níveis similares de cálcio, fosfato e paratormônio. Quando a função renal diminuiu, a porcentagem de discordância entre os ensaios aumentou.

**Conclusão:**

Existe uma notável variabilidade entre os ensaios em pacientes com DRC, de modo a modificar o diagnóstico de hipovitaminose D. O mecanismo por trás desse resultado ainda não está claro e pode ser devido a uma possível interferência no processo analítico. Entretanto, o significado clínico é inquestionável, pois a suplementação de vitamina D pode ser erroneamente prescrita a esses pacientes.

## Introduction

Several studies have suggested that the effects of vitamin D are not limited to the skeleton and muscles, providing additional clinical benefits.^1^ Patients who are screened for vitamin D deficiency usually receive ergocalciferol (D2) or cholecalciferol (D3) supplementation.^1^

Clinical recommendations for the screening and treatment of vitamin D deficiency are highly variable and controversial. However, there is a consensus that patients with chronic kidney disease (CKD) should receive supplementation since they have an increased risk of hypovitaminosis D and secondary hyperparathyroidism.^1^^,^ 2

The inter-assay variability is a confounding factor that challenges the precision of 25-hydroxyvitamin D measurement. Since the gold standard method (high performance liquid chromatography - HPLC) is considered a cumbersome assay, other options are used instead^3^^,^ 4. Usually, the validation of new 25-vitamin D assays is done through the comparison with HPLC results.^5^^-^ 7 However, the CKD population is either not well represented or excluded from these studies.^2^^,^ 4 ^,^ 8 ^-^ 10

In this study, we investigated the impact of various stages of CKD on the variability between two chemiluminescence assays for 25-vitamin D.

## Patients and methods

### Source population and data collection

This was a retrospective study to compare two 25-vitamin D assays measurements with patients from a nephrology outpatient clinic located at Hospital das Clinicas, Universidade de Sao Paulo, Brazil.

Data were obtained from electronic charts. We screened a total of 991 patients who had two consecutive 25-vitamin D measurements, one in 2015 (period 1) and one in 2016 (period 2). In 2015, serum 25-vitamin D was measured using the Diasorin LIASON 25 TOTAL - D assay^®^ (Assay 1), and in 2016 it was measured with the Beckman Coulter Unicel Xl 800^®^ (Assay 2). We excluded kidney transplant recipients (n = 121) and patients with missing data on variables of interest (n = 79), which included calcium, phosphate, PTH, and estimated glomerular filtration rate (eGFR). The remaining 791 patients were divided according to the renal function, based on eGFR calculated by CKD-EPI equation. Patients were classified by CKD stage and the results of assay 1 and assay 2 were compared. Figure S1 summarizes this flowchart.

Data collected included age, gender, serum calcium (Ca; reference range [RR] = 4.6-5.3 mg/dL), serum alkaline phosphatase (AP; RR = 32-129 U/L), serum phosphate (P; RR = 2.7- 4.5 mg/dL), intact parathyroid hormone (PTH; RR 15-65 pg/mL), and serum 25-vitamin D obtained with assays 1 and 2 - by chemiluminescence method. We also collected data on cholecalciferol supplementation (UI/week). Normal levels of 25-vitamin D were defined as equal or higher than 30 ng/mL, whereas hypovitaminosis was considered when serum levels were below 30 ng/mL.

The local Reseach Ethics Comittee approved this study (CAPPESQ #45163715.4.0000.0068).

### Assays for 25-vitamin D measurements

The assay 1, used in the first period of evaluation, is a chemiluminescent immunoassay that quantifies 25-hydroxyvitamin D and other hydroxylated vitamin D metabolites in human serum. It is a direct competitive chemiluminescence immunoassay (CLIA). The unit of measure is ng/mL and its measurement range is 4-150 ng/mL. The assay 2, used in the second period of evaluation, is a two-step competitive binding immunoenzymatic assay that uses a 25-hydroxyvitamin D analogue - alkaline phosphatase conjugate - which is added and competes for binding to the immobilized monoclonal anti-25-hydroxyvitamin D.^11^ Results range from 7.0 to 120 ng/mL. The amount of analyte in the sample is determined from a stored multi-point calibration curve.

### Statistical analysis

Data are reported as mean ± SD or median and 25 and 75 percentiles for normally and non-normally distributed variables, respectively. Comparisons among groups according to renal function were done by ANOVA or Kruskal-Wallis according to data distribution. We used Student t-test or Mann-Whitney test to compare variables between assays 1 and 2. To compare categorical variables we used Chi-square or Fisher test, as appropriate. The comparisons between the two assays for measurement of 25-vitamin D were performed utilizing a Bland-Altman approach. We used SPSS 21.0 (SPSS Inc.,Chicago IL) and GraphPad Prism 6 Software (GraphPad Software Inc., San Diego, CA, USA) for statistical analyses.

## Results

Table 1 shows the characteristics of patients classified according to eGFR. Patients with eGFR > 90 mL/min/1.73m^2^ were younger and had lower levels of PTH, whereas those on dialysis had lower serum Ca, and higher P and PTH. Supplementation and doses of vitamin D_3_ were similar among groups and between assays 1 and 2. However, the 25-vitamin D levels detected by assay 2 were higher than those by assay 1, with no differences in P, Ca, and PTH levels between the two periods. This difference was significant only for patients with an eGFR below 60 mL/min/1.73m^2^.

**Table 1:** Characteristics of patients according to estimated glomerular filtration rate (eGFR) using assays 1 and 2 to measure 25 vitamin D

eGFR, mL/min/1.73m^2^	ALL N = 791	> 90 N = 122	30-60 N = 221	15-30 N = 198	ESRD on dialysis N = 250
Age (years)	57 ± 19	39 ± 14^a^	62 ± 16	66 ± 15	53 ±18
Male (%)	49.5	33.0 ^a^	56.0	52.0	50.0
Assay 1					
Ca (mg/dL)	9.3 ± 0.6	9.2 ± 0.6	9.4 ± 0.5 ^b^	9.4 ± 0.4	9.2 ± 0.7 ^b^
P (mg/dL)	3.5 ± 0.6	3.5 ± 0.6	3.4 ± 0.6	3.6 ± 0.6	3.9 ± 0.7 ^a^
PTH (pg/mL)	75 (46; 121)	30 (23; 43) ^a^	58 (44; 79) ^a^	99 (67; 151) ^a^	125 (98; 191) ^a^
25 vit D (ng/dL)	23.9 ± 8.9	23.3 ± 10.2	24.0 ± 8.4	23.9 ± 8.5	24.0 ± 9.1
Vit. D_3_ supplementation					
Use (%)	52.3	57.4	44.8	54.0	55.2
Doses (1,000 UI/week)	12.5 (10.0; 20.0)	13.3 (10.0; 20.0)	12.0 (10.0; 15.0)	12.5 (10.0; 20.0)	12.5 (10.0; 20.0)
Assay 2					
Ca (mg/dL)	9.3 ± 0.6	9.3 ± 0.6 ^c^	9.6 ± 0.6 ^a^	9.3 ± 0.5	9.0 ± 0.6 ^a^
P (mg/dL)	3.6 ± 0.7 ^c^	3.5 ± 0.6	3.4 ± 0.6	3.6 ± 0.6	4.3 ± 0.8 ^a^
PTH (pg/mL)	82 (53; 134)	33 (24; 41) ^a^	61 (48; 84) ^a^	104 (80; 142) ^a^	177 (119; 252) ^a^
25 vit D (ng/dL)	30.0 ± 12.3 ^c^	23.9 ± 9.0 ^a^	30.2 ± 10.9 ^c^	30.9 ± 11.1 ^c^	32.0 ± 14.8 ^c^
Vit.D_3_ supplementation					
Use (%)	54.5	65.6	48.9	59.1	50.4
Doses (1,000 UI/week)	12.5 (8.0; 20.0)	12.5 ((10.0; 20.0)	12.5 (10.0; 20.0)	12.0 (7.0; 20.0)	14.0 (10.0; 25.0)

a *p* < 0.05 *vs*. all other groups;

b *p* < 0.05 *vs*. > 90 and 30-60;

c *p* < 0.05 *vs*. the same variable using Assay 1.

**Figure 1:**
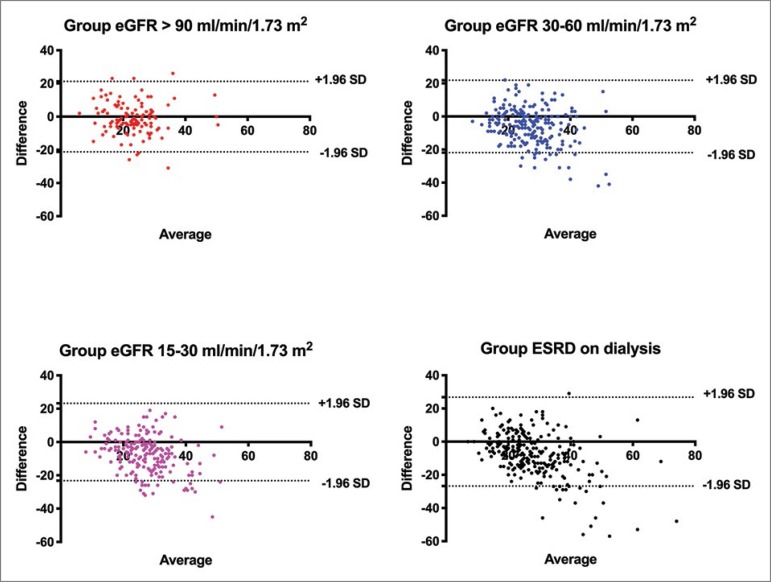
Bland Altman plot of agreement between 25 vitamin D measured by assay 1 and assay 2. Horizontal dotted lines represent limits of agreement (i.e., 1.96 standard deviations of inter-modality difference). Bias were -0.6 ± 10.6, -6.1 ± 10.9, -7.0 ± 11.6, and -7.9 ± 13.4 in groups eGFR > 90, 30-60, 15-30 mL/min/1.73m^2^, and ESRD on dialysis, respectively. Limits of agreement were -21.4 to -20.2, -27.6 to -15.3, -29.8 to -15.7, and -34.2 to -18.3 in groups with eGFR > 90, 30-60, 15-30 mL/min/1.73m^2^ and ESRD on dialysis, respectively.

The prevalence of hypovitaminosis D was higher using assay 1 in patients with eGFR lower than 60mL/min/1.73m^2^ and ESRD on dialysis (Figure S2). The percentage of patients classified as having normal or low levels of 25-vitamin D using assays 1 and 2 is plotted in Table S1. Of note, as the eGFR decreases, the percentage of disagreement between the assays increases. Bland-Altman plots showing agreement between assays 1 and 2 are depicted in Figure 1. Limits of agreement were narrowest in patients with eGFR > 90 mL/min/1.73m^2^.

## Discussion

The results of this study show a disagreement between the two assays for measurement of 25-vitamin D in patients with CKD, indicating that a relatively significant proportion of patients were misclassified as having hypovitaminosis D or normal 25-vitamin D status. The lowest bias was obtained in patients with normal renal function (eGFR > 90 mL/min/1.73m^2^), suggesting the presence of some unidentified interference in the CKD population that might impact the assays’ results.

This study was motivated by the general observation of higher levels of 25-vitamin D in the Nephrology service, despite no changes in both the routine supplementation of cholecalciferol and the levels of calcium, phosphate, and PTH. With this in mind, we were informed about the new assay that was being used. In addition, if the levels of 25-vitamin D were really higher as the second assay showed, why the PTH did not decrease?^1^

The question being asked in this comparison is whether either of the two assays could be used to measure 25-vitamin D levels evenly. To answer this, 25-vitamin D must be measured at the same time with simultaneous sampling. The measurements with the tested assays done months apart is certainly a limitation of our study. However, since the levels of calcium, phosphate, PTH, and the supplementation of vitamin D remained unchanged between the two measurements suggests levels of 25-vitamin D should be also similar.

Nevertheless, part of the difference between measurements of 25-vitamin D can be explained by season of the year (summer vs winter). However, this is unlikely to have happened in our study since the second assay was performed during the winter (less sun exposure) and still higher levels of 25-vitamin D were found.

Therefore, our main hypothesis relied upon the analytical process. As mentioned before, the CKD population is usually excluded from trials, and studies comparing assays with HPLC in this population are lacking. Our findings highlighted that the disagreement was mainly in patients with eGFR < 60mL/min/1.73m^2^. We hypothesized that: 1. Non-active fragments, such as urea or other retained metabolites, present in this population might be recognized as intact 25-vitamin D and affect the assay performance^3^^,^ 8 ^,^ 12 ^-^ 14; 2. Because assay 2 uses analogue-alkaline phosphatase conjugate, which competes for binding to the monoclonal anti-25-vitamin D before being read by the luminometer and CKD patients usually present higher endogenous alkaline phosphatase, this works as an interference and might cause a false positive^15^; 3. Assay 2 should not be used in patients using paricalcitol, which is also very common in this population. However, in our sample, none of the patients was using this drug.

In short, we found a disagreement between the two assays for 25-vitamin D measurements, which is of clinical importance in patients with CKD. In this population, we should make efforts to develop assay standardization. It is not clear why there is such disagreement between assays. Nevertheless, clinicians should be alert of the limitations of the immunoassays and interpret the results cautiously to avoid misinterpretation and erroneous prescription of vitamin D supplementation.

## Supplementary material

“The following online material is available for this article:

**Figure S1** - Flow chart of patients included in the study.

**Figure S2** - Prevalence of hypovitaminosis D according to renal function (eGFR > 90 or < 60 ml/min/1.73m^2^).

**Table S1** - Comparison between normal and low serum 25-vitamin D prevalence between assays 1 and 2
