# Supplementary material for: Chronic kidney disease is a main confounding factor for 25-vitamin D measurement
Source: J Bras Nefrol. 2019 Sep 26;42(1):94–8. doi: 10.1590/2175-8239-JBN-2019-0053 (PMC7213929; doi:10.1590/2175-8239-JBN-2019-0053)
Supplement: Supplementary file 2 [file 2175-8239-jbn-2019-0053-suppl2.pdf]

Supplementary Material to "Chronic Kidney Disease is a main confounding factor for 25-vitamin D measurement"

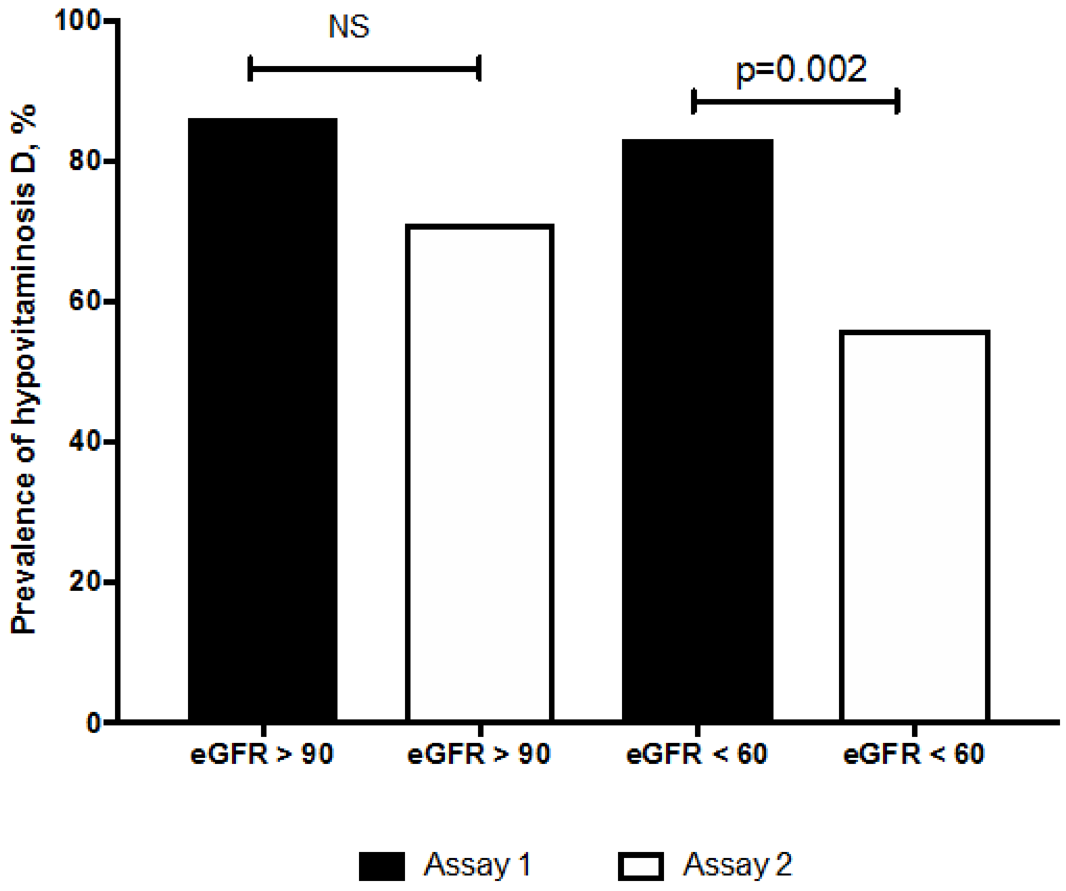

Figure S2 - Prevalence of hypovitaminosis D according to renal function (eGFR > 90 or <60 mL/min/1.73m^2) detected by assays 1 and 2.
