# Supplementary material for: Chronic kidney disease is a main confounding factor for 25-vitamin D measurement
Source: J Bras Nefrol. 2019 Sep 26;42(1):94–8. doi: 10.1590/2175-8239-JBN-2019-0053 (PMC7213929; doi:10.1590/2175-8239-JBN-2019-0053)
Supplement: Supplementary file 3 [file 2175-8239-jbn-2019-0053-suppl3.pdf]

Supplementary Material to "Chronic Kidney Disease is a main confounding factor for 25-vitamin D measurement"

Table S1 - Comparison between normal and low serum 25-vitamin D prevalence measured with assays 1 and 2.

	**Assay 1**	**Assay 2**
	Normal 25-vitamin D	Hypovitaminosis D
**Control**		
Normal 25-vitamin D	8.2	9.8
Hypovitaminosis D	15.6	66.4
**eGFR 30-60**		
Normal 25-vitamin D	16.7	7.7
Hypovitaminosis D	34.0	41.6
**eGFR 15-30**		
Normal 25-vitamin D	12.6	8.6
Hypovitaminosis D	38.9	39.9
**ESRD on Dialysis**		
Normal 25-vitamin D	16.9	4.4
Hypovitaminosis D	35.7	43.0

Numbers in grey indicate percentage of agreement between assays.
